# Supplementary material for: Solvent-dripping modulated 3D/2D heterostructures for high-performance perovskite solar cells
Source: Nat Commun. 2025 Jan 26;16:1042. doi: 10.1038/s41467-025-56409-5 (PMC11763036; doi:10.1038/s41467-025-56409-5)
Supplement: Supplementary file 1 — Supplementary Information [file 41467_2025_56409_MOESM1_ESM.pdf]

Supplementary information for

Solvent-dripping modulated 3D/2D heterostructures for high-performance perovskite solar cells

Xiaoming Chang[1,#], Randi Azmi[2,#], Tinghuan Yang[3], Nan Wu[3], Sang Young Jeong[4], Herui Xi[5], Drajad Satrio Utomo[1], Badri Vishal[1], Furkan H. Isikgor[1], Hendrik Faber[1], Zhaoheng Ling[1], Mingjie He[1], Marco Marengo[1], Pia Dally[1], Adi Prasetio[1], Yu-Ying Yang[1], Chuanxiao Xiao[5,6], Han Young Woo[4], Kui Zhao[3], Martin Heeney[1], Stefaan De Wolf[1], Leonidas Tsetseris[7], Thomas D. Anthopoulos[1,8,*]

[1]KAUST Solar Center (KSC), Physical and Engineering Division (PSE), King Abdullah University of Science and Technology (KAUST), Thuwal 23955-6900, Kingdom of Saudi Arabia.

[2]School of Science and Engineering, The Chinese University of Hong Kong, Shenzhen, Guangdong 518172, China

[3]School of Materials Science and Engineering, Shaanxi Normal University, Xi'an 710119, China.

[4]Department of Chemistry, Korea University, Anam-ro 145, Seoul 02841, Republic of Korea.

[5]Ningbo Institute of Materials Technology and Engineering, Chinese Academy of Sciences, Ningbo City, China.

[6]Ningbo New Materials Testing and Evaluation Center Co., Ltd, Ningbo City, China

[7]Department of Physics, School of Applied Mathematical and Physical Sciences, National Technical University of Athens, Athens GR-15780, Greece.

[8]Henry Royce Institute, Photon Science Institute, Department of Electrical and Electronic Engineering, The University of Manchester, Manchester M13 9PL, UK.

[#]These authors contributed equally: Xiaoming Chang, Randi Azmi.

*Corresponding authors: thomas.anthopoulos@manchester.ac.uk

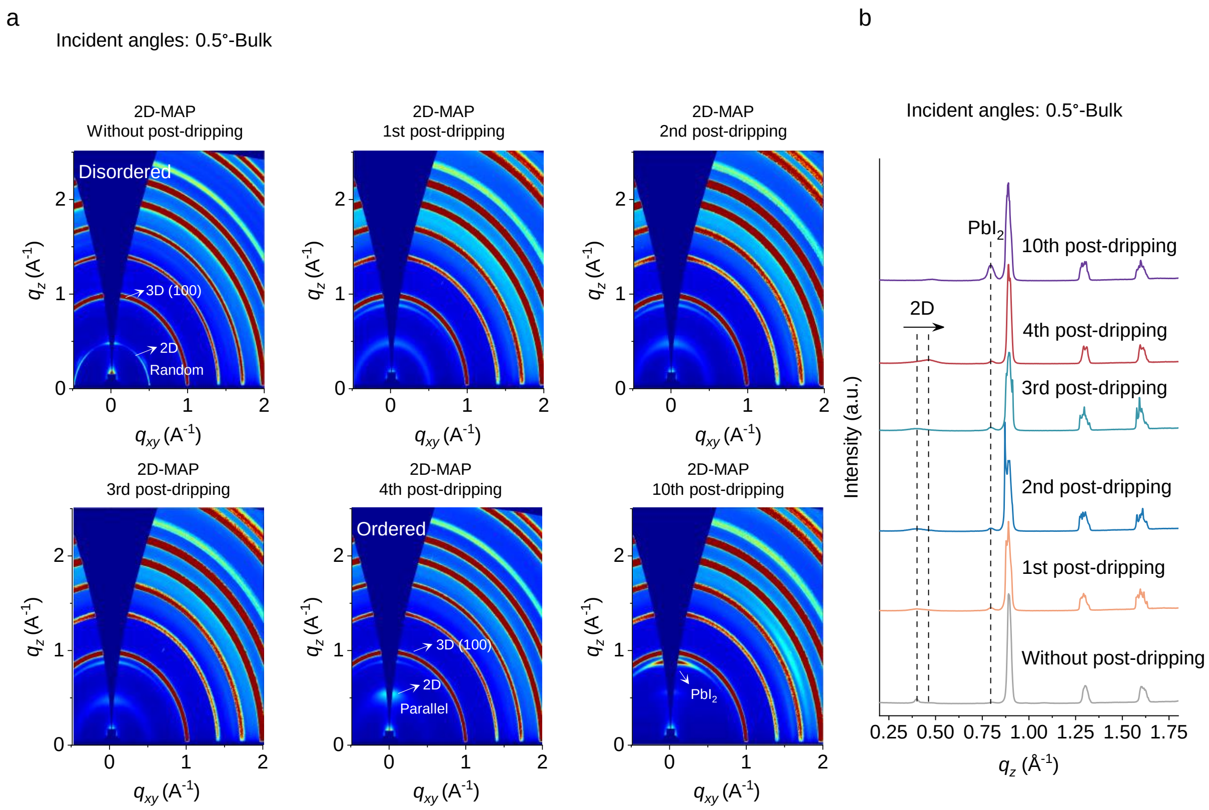

Fig. S1: **a**, 2D GIWAXS images of perovskite films for different post-dripping time with 0.5° X-ray incidence angles. **b**, Combined intensity from GIWAXS data in the q_z direction with 0.5° X-ray incidence angles.

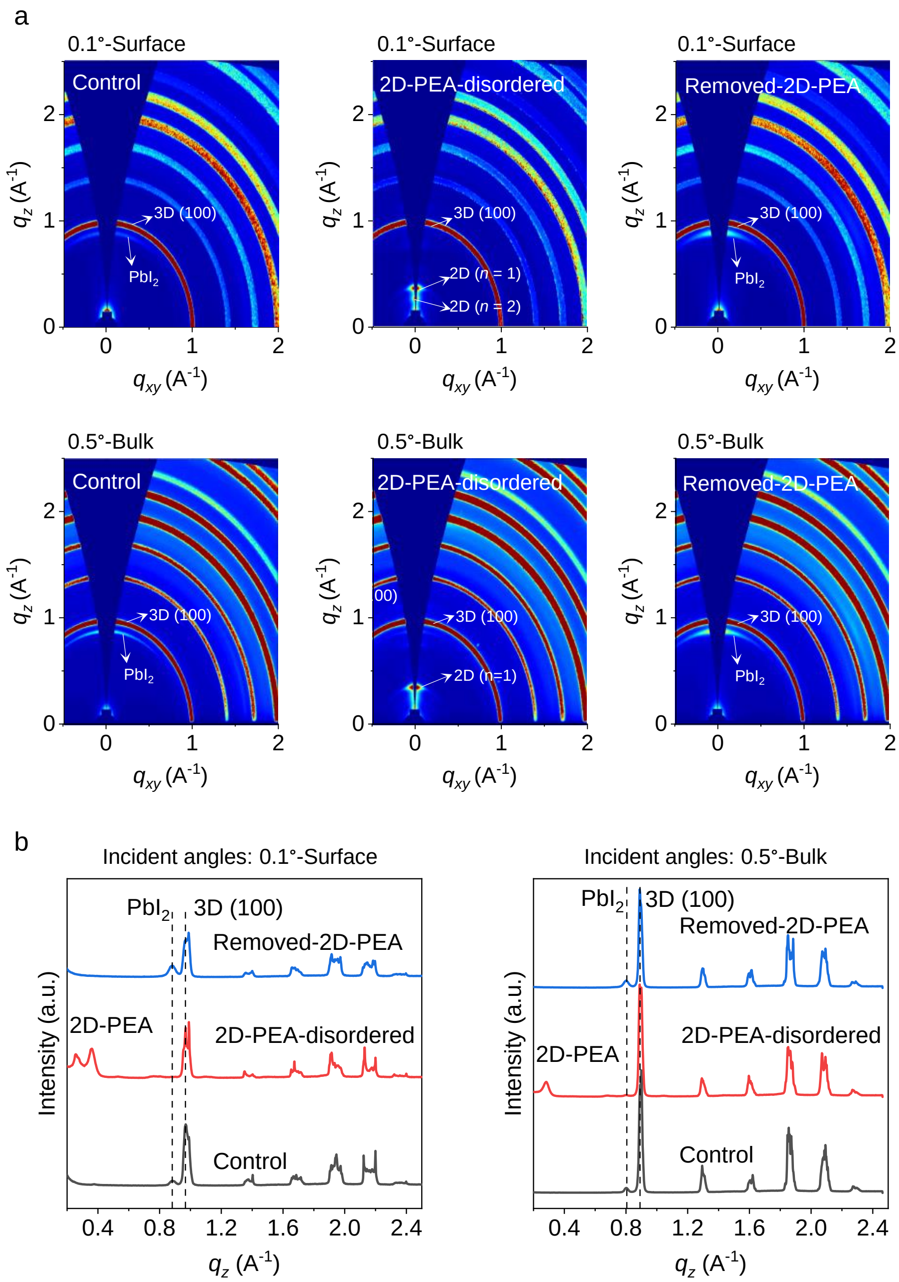

Fig. S2: a, 2D GIWAXS images of perovskite films control, 2D-PEA-disordered and removed-2D-PEA with 0.1° and 0.5° X-ray incidence angles. **b**, Combined intensity from GIWAXS data in the q_z direction with 0.1° and 0.5° X-ray incidence angles.

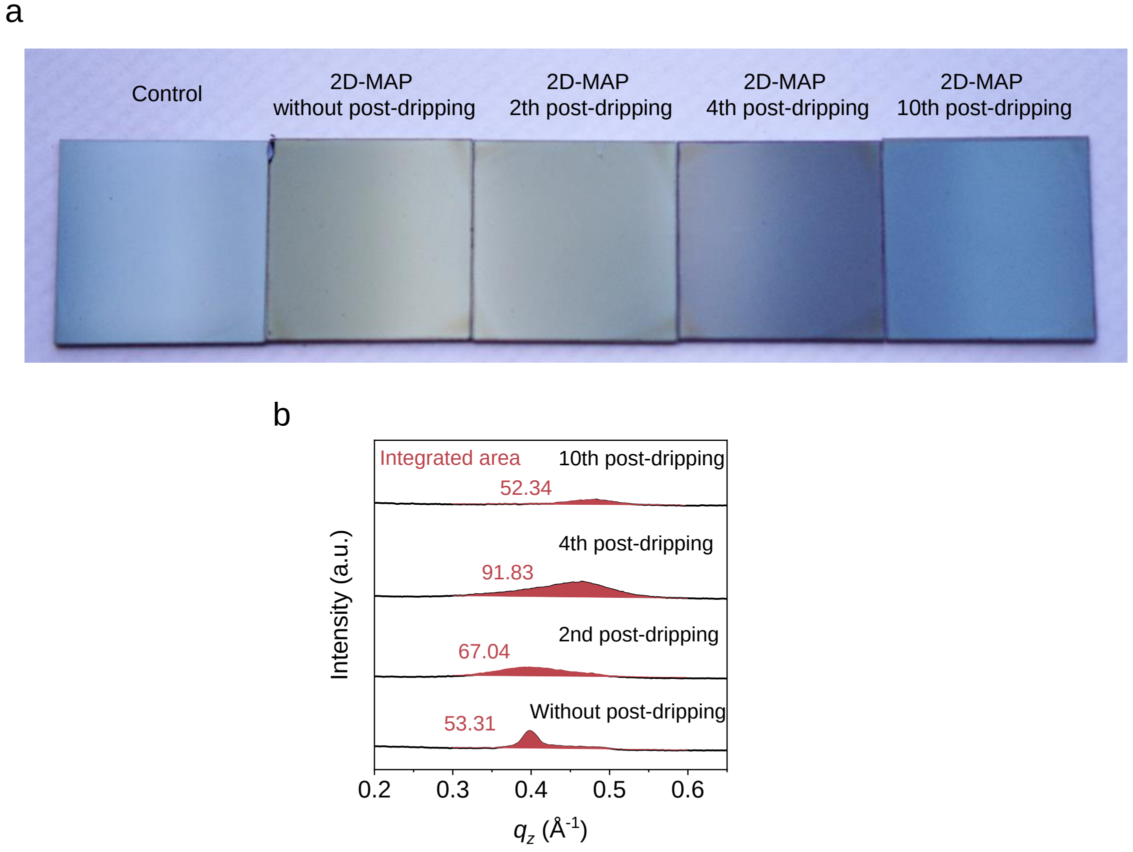

Fig. S3: a, Photos of perovskite films made on ITO glass substrates. **b,** The integrated area of 2D peak for different IPA post-dripping 3D/2D perovskite films.

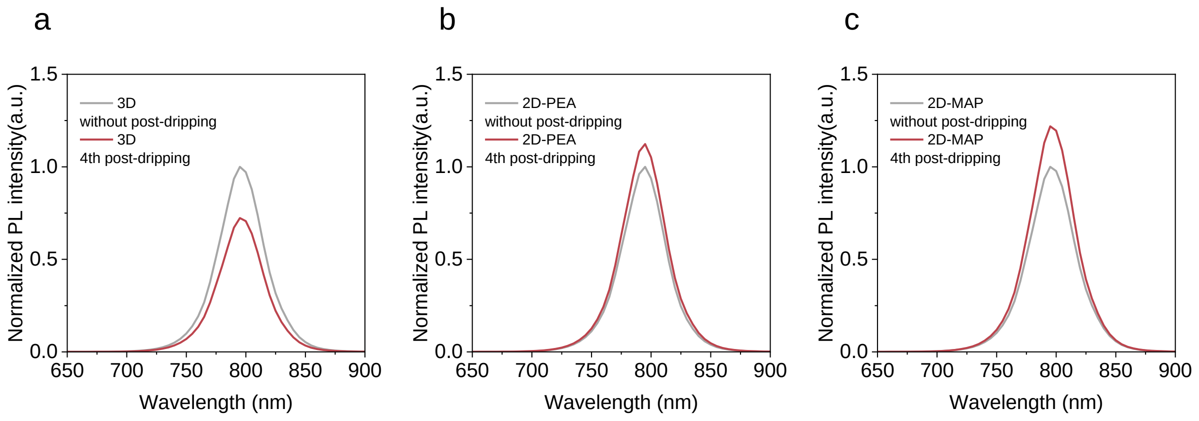

Fig. S4: a-c, Comparison of PL spectra for 3D, 2D-PEA-treated, and 2D-MAP-treated perovskite films.

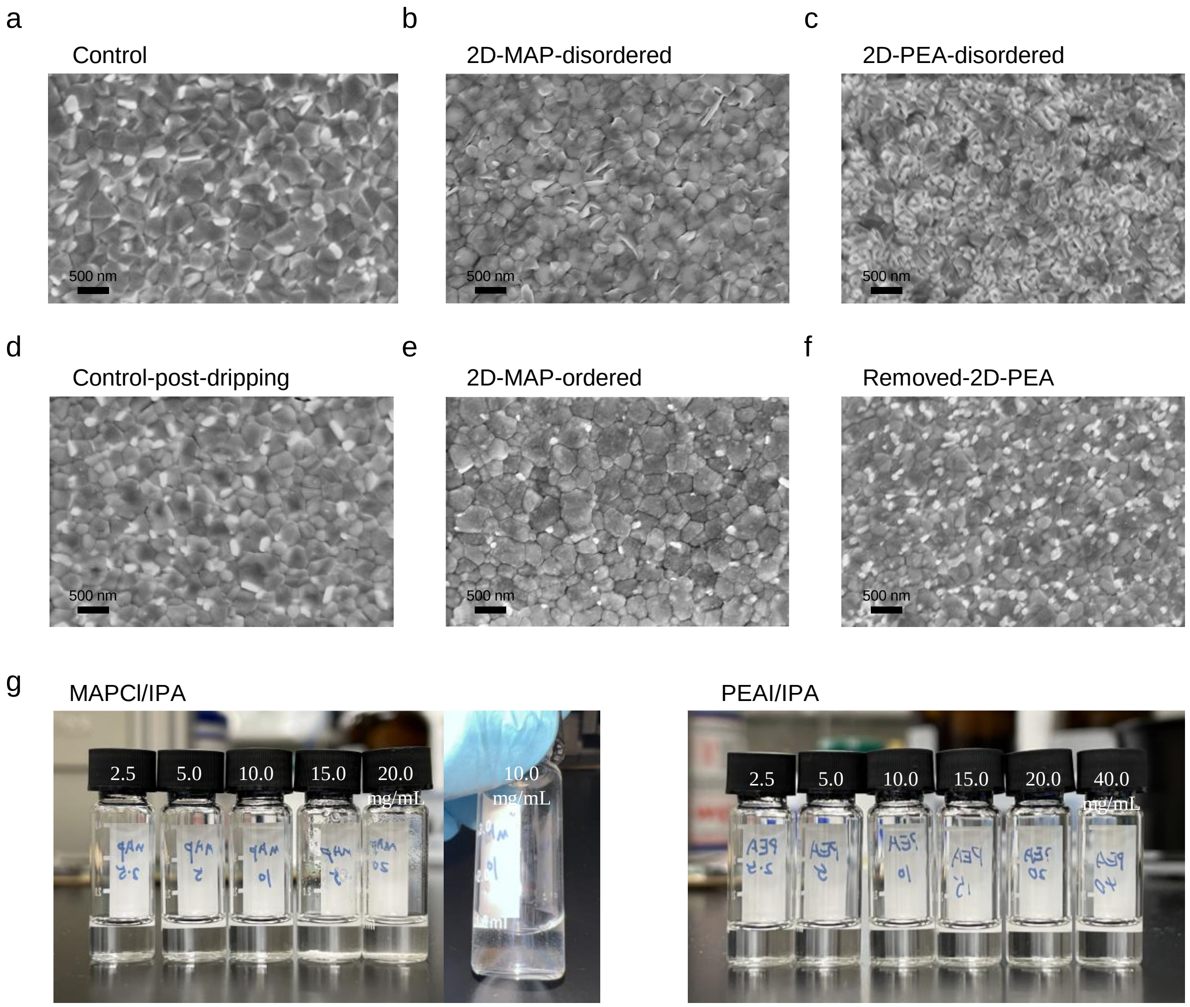

Fig. S5: a-f, SEM images of perovskite films with or without IPA post-dripping steps. **g**, Solubility test of PEAI and MAPCl ligands in IPA.

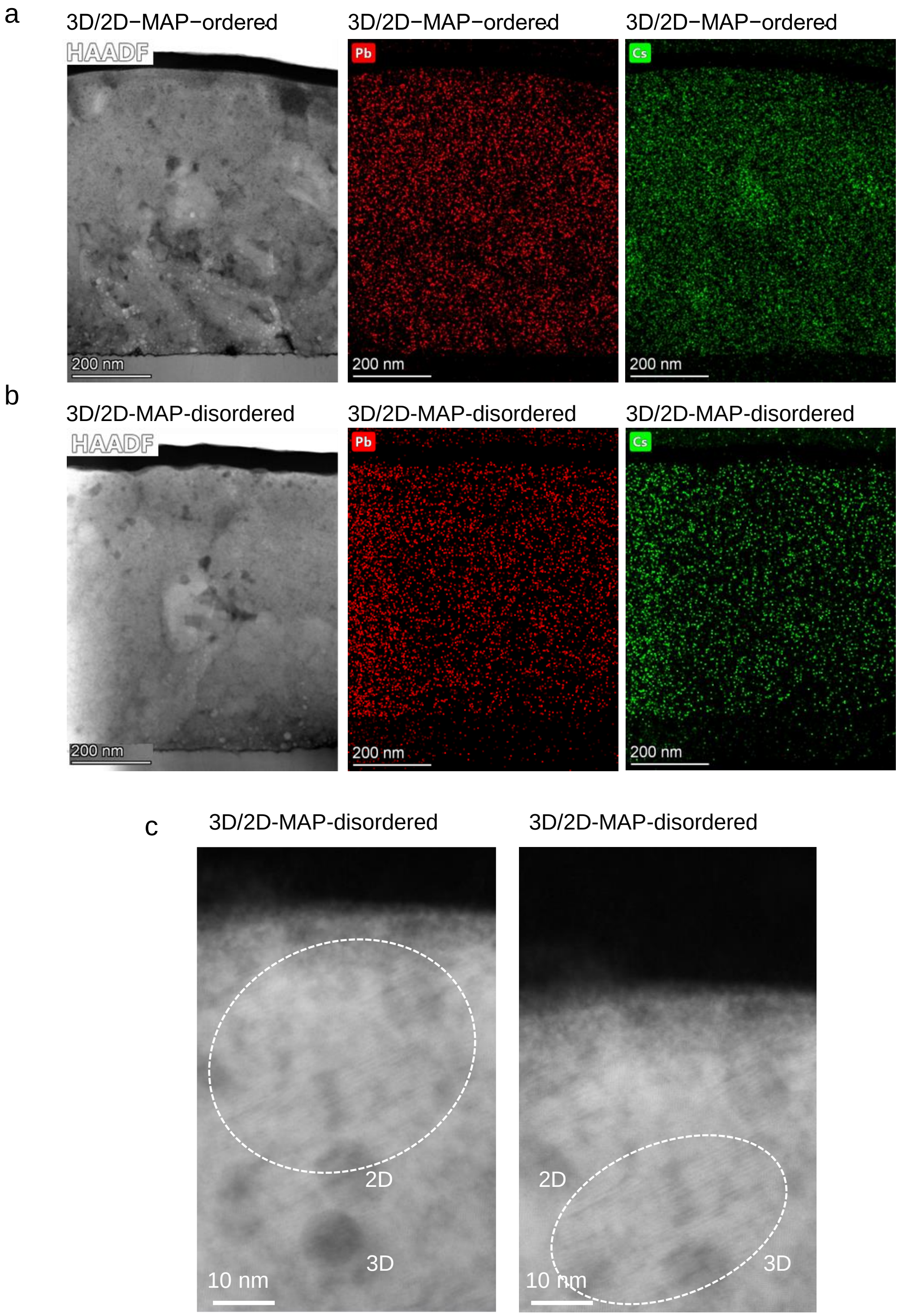

Fig. S6: Cross-sectional HR-STEM and EDX images of top 3D/2D interface of devices (**a**, 2D-MAP-disordered and **b** 2D-MAP-ordered). **c**, Cross-sectional HR-STEM of 2D-MAP-disordered.

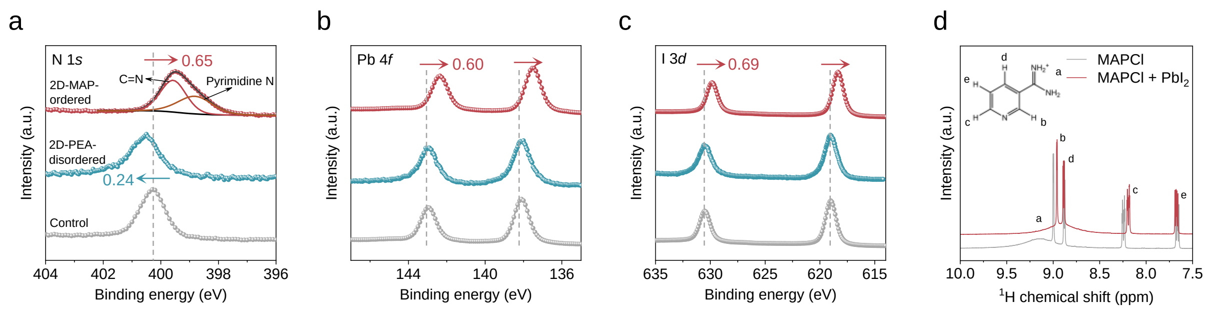

Fig. S7: a-c, XPS spectra (N 1*s*, Pb 4*f*, and I 3*d*) obtained from the different perovskite layers. **d**, ^{1}H NMR spectra of MAPCl and MAPCl + PbI_2.

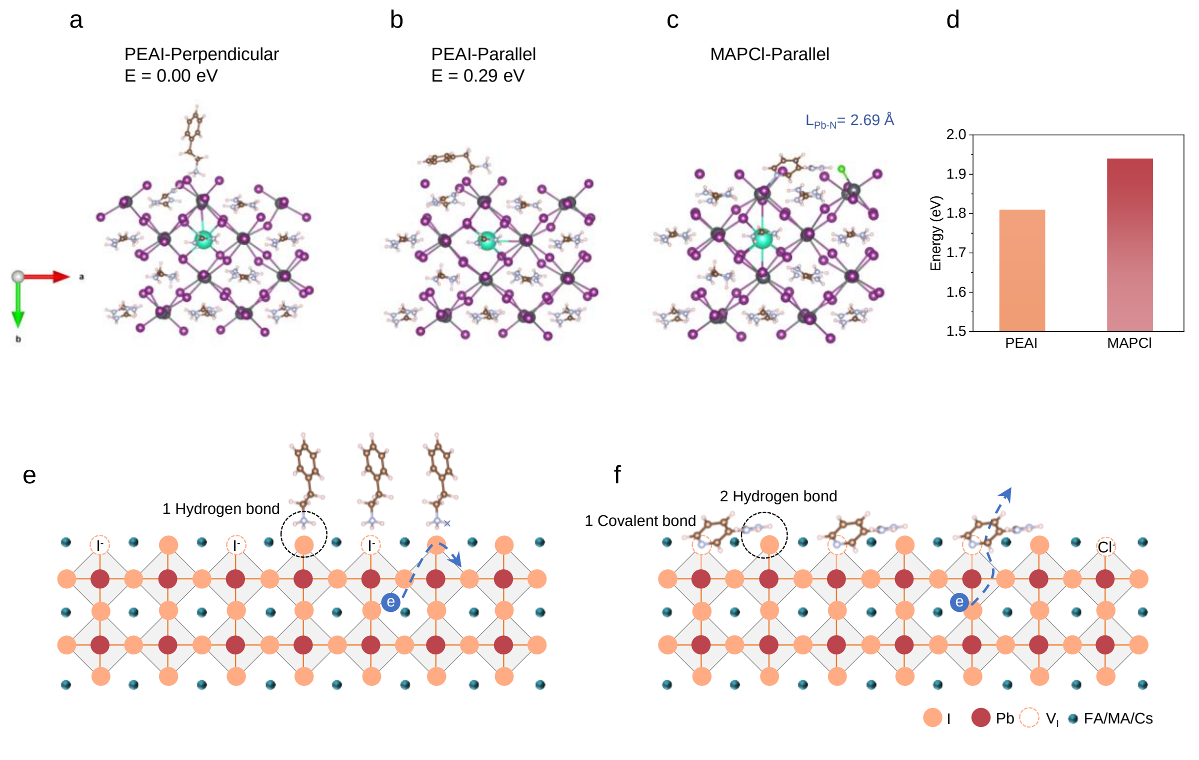

Fig. S8: a-c, Adsorption of PEAI and MAPCl on the (110) surface of $FAMACsPbI_3$ (C: Reddish-brown, N: Light steel blue, H: Pastel pink, Pb: Dark gray, I: purple, Cs: Bright turquoise, and Cl: Green spheres). E is the relative energy of the structure. It is energetically favorable for the PEA^+ cations to remain upright as in (**a**), while the corresponding I^- anions passivates the surface I-vacancy sites. **d**, Surface adsorption energy of PEAI and MAPCl. **e**,**f**, Schematic of the perovskite surface with PEAI and MAPCl passivation.

We assessed ligand passivation by analyzing iodide vacancies, the primary defects on the perovskite surface. **Fig. S8a-c** illustrates the interaction between PEAI and MAPCl with the 3D perovskite, as revealed by Density Functional Theory (DFT) calculations. The calculations show that PEA and MAP absorb protons from HI and HCl complexes, becoming positively charged, while iodide (I^-) and chloride (Cl^-) anions fill surface vacancies. Both PEA^+ and MAP^+ form hydrogen bonds with halogen sites through their terminal groups. MAP^+ contains more N-H bonds, increasing its interaction with the PbI_6 octahedra, and MAP^+ also forms covalent bonds with surface Pb atoms for added stability. The adsorption of PEA^+ and MAP^+, combined with the surface vacancy passivation by I^- and Cl^-, leads to significant energy gains of 1.81 eV (PEAI) and 1.94 eV (MAPCl) (**Fig. S8d**).

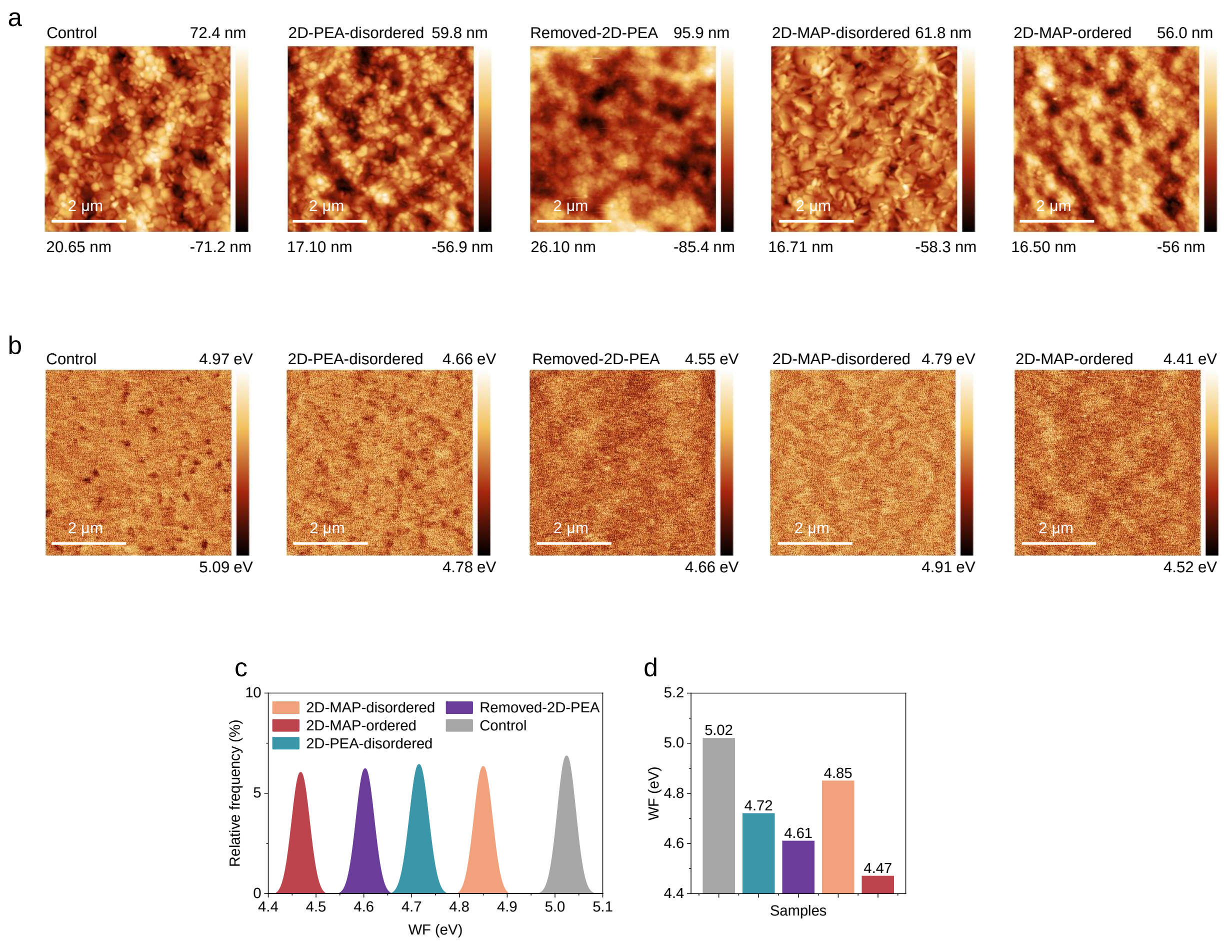

Fig. S9: a, AFM morphology images of perovskite films. **b**, KPFM surface potential images of perovskite films. **c**, Histograms of the work function distributions obtained via KPFM. **d**, The work function comparison of perovskite films.

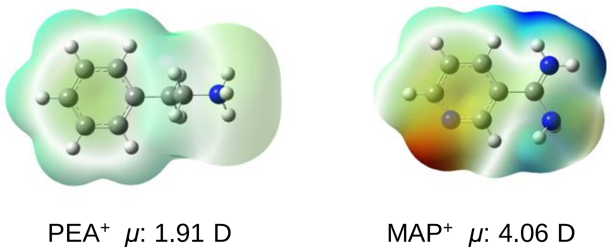

Fig. S10: Structure and electrostatic potential of PEA^+ and MAP^+ cations.

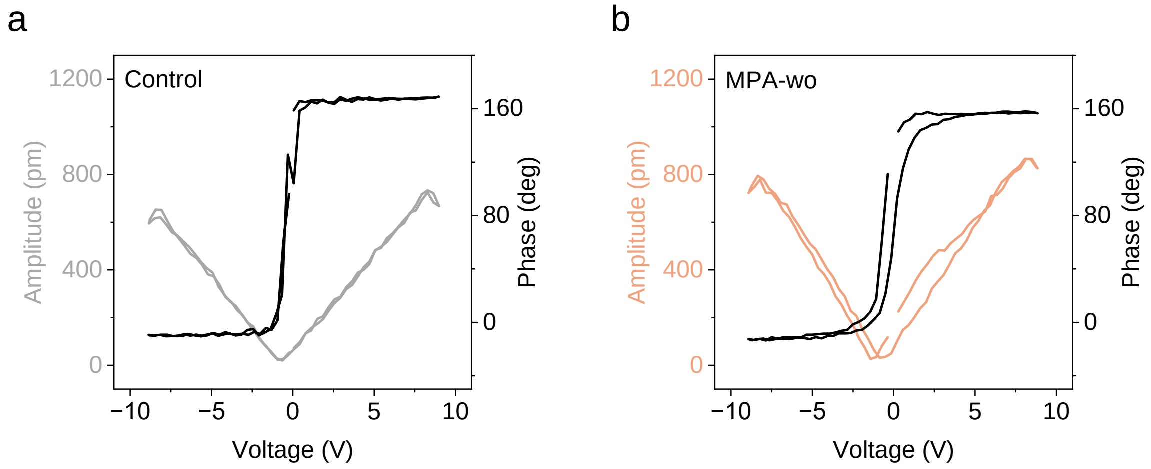

Fig. S11: **a**,**b**, Hysteretic dependence of the PFM phase and amplitude with the applied DC bias for perovskite films.

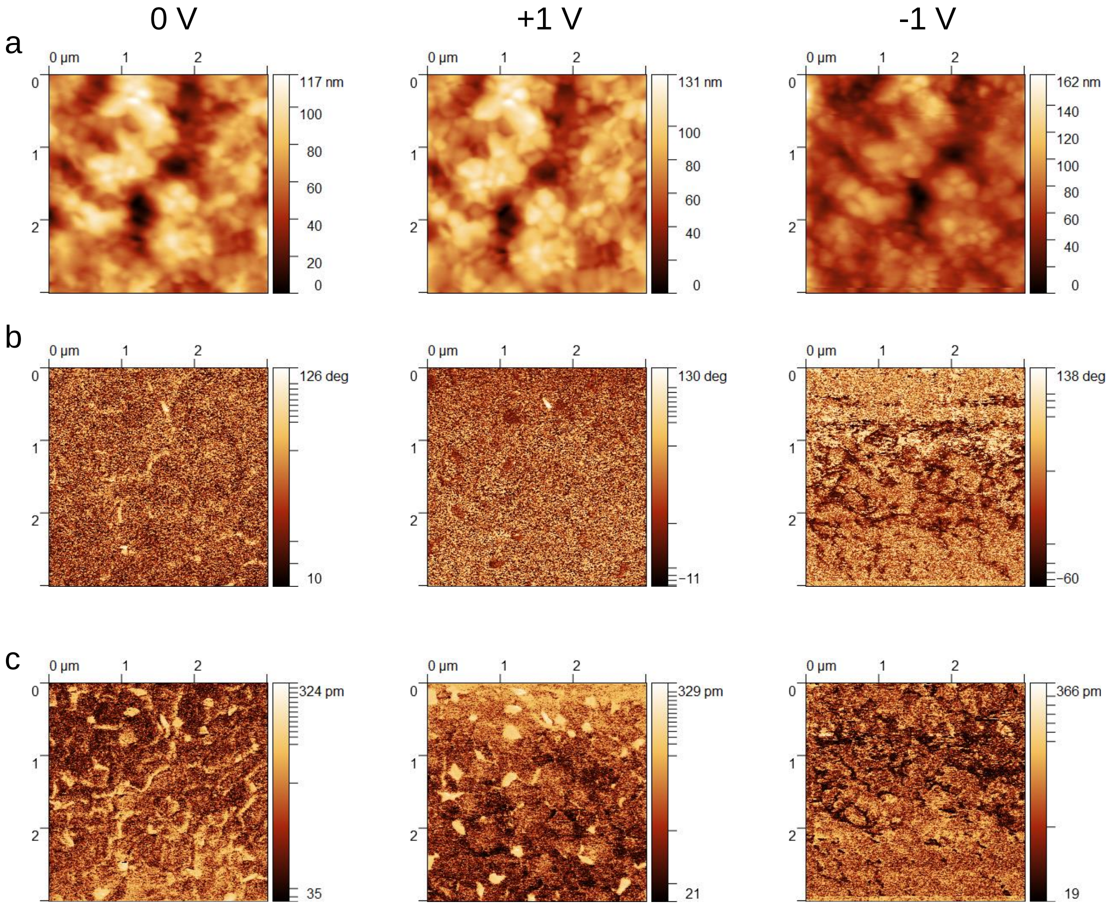

Fig. S12: a, AFM images of the control perovskite films under different bias voltages. **b**, PFM phase images of the control perovskite films under different bias voltages. **c**, PFM amplitude images of the control perovskite films under different bias voltages.

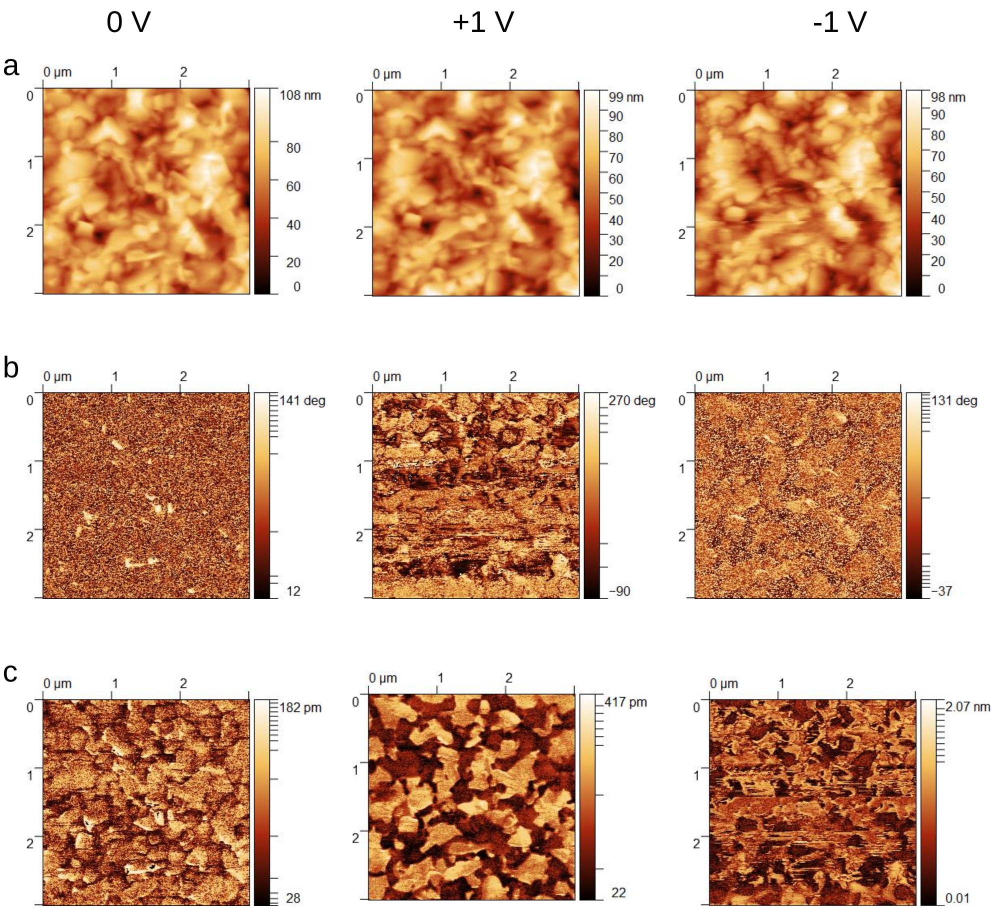

Fig. S13: a, AFM images of the 2D-MAP-disordered-based perovskite films under different bias voltages. **b**, PFM phase images of the 2D-MAP-disordered-based perovskite films under different bias voltages. **c**, PFM amplitude images of the 2D-MAP-disordered-based perovskite films under different bias voltages.

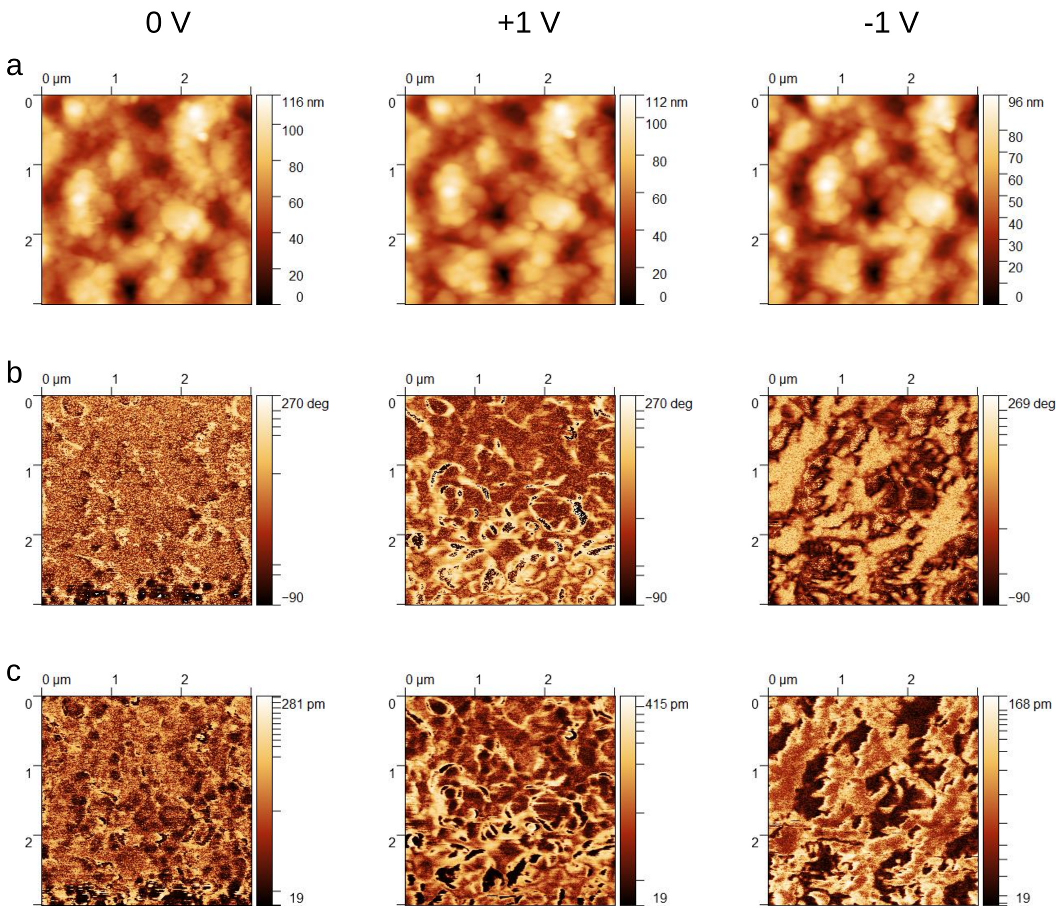

Fig. S14: a, AFM images of the 2D-MAP-ordered-based perovskite films under different bias voltages. **b**, PFM phase images of the 2D-MAP-ordered-based perovskite films under different bias voltages. **c**, PFM amplitude images of the 2D-MAP-ordered-based perovskite films under different bias voltages.

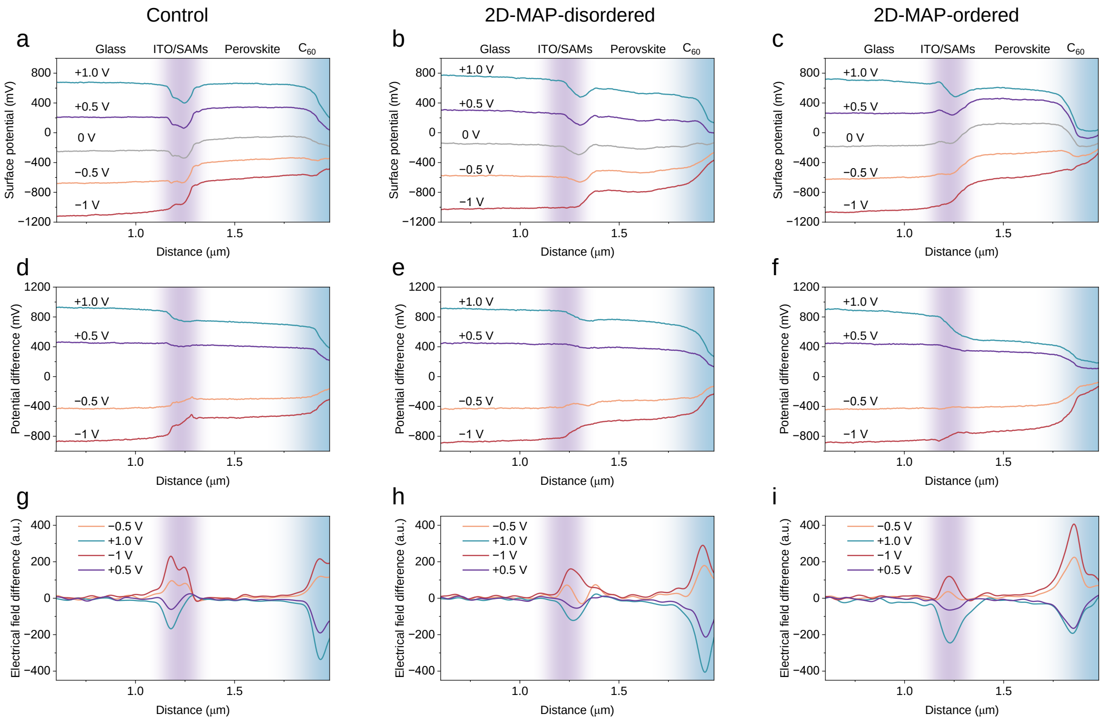

Fig. S15: a-c, Surface potential depth profiles under different bias voltages. **d-f**, Potential differences depth profiles subtracted by the 0 V curve. **g-i**, Electric-field difference depth profiles, taken by the first derivative in (**d-f**).

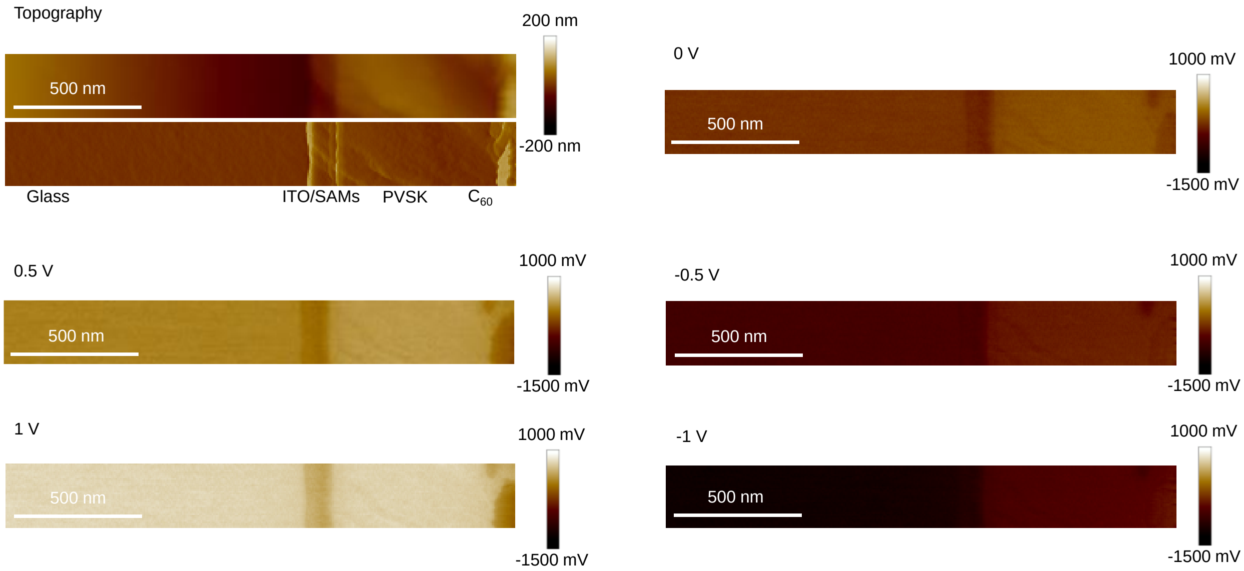

Fig. S16: AFM topography images and corresponding KPFM images under different bias voltages for control-based devices.

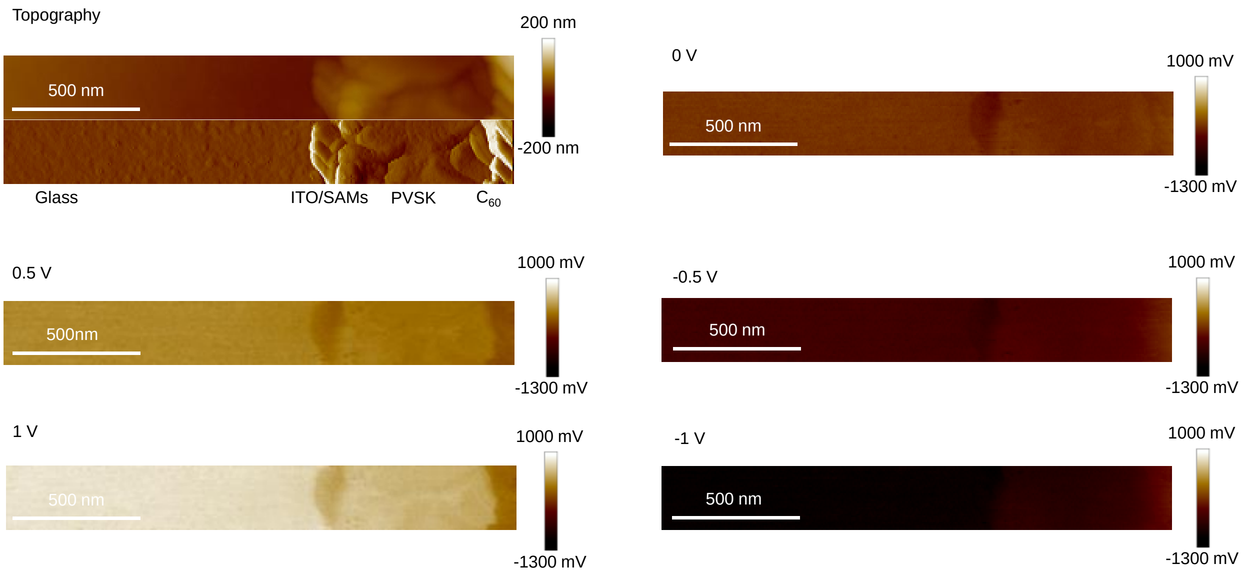

Fig. S17: AFM topography images and corresponding KPFM images under different bias voltages for 2D-MAP-disordered-based devices.

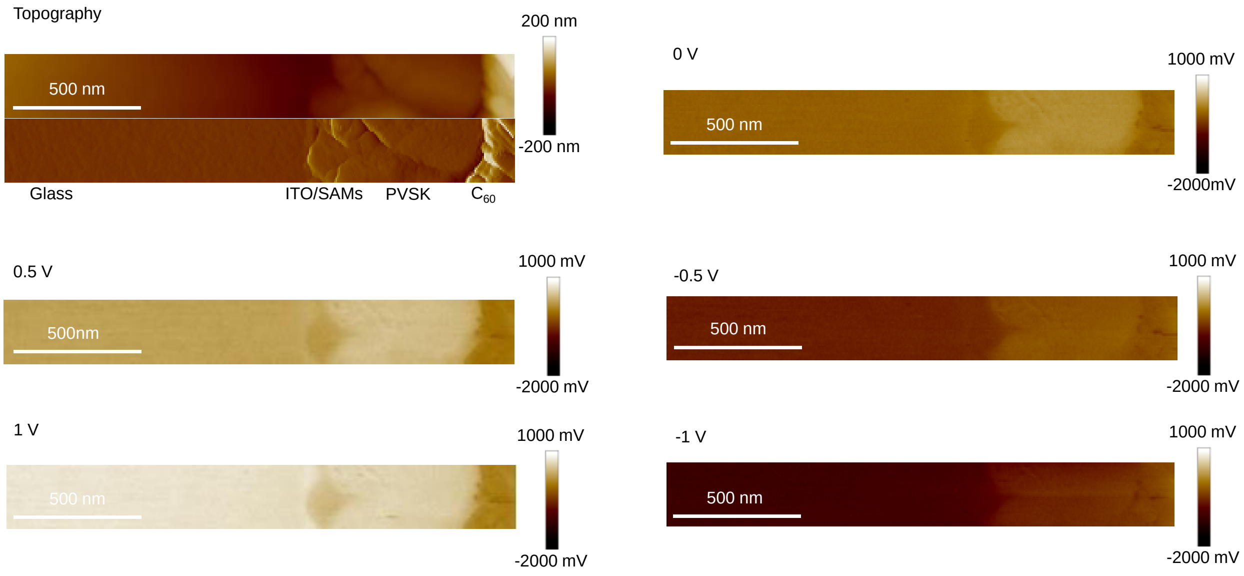

Fig. S18: AFM topography images and corresponding KPFM images under different bias voltages for 2D-MAP-ordered-based devices.

中国认可
检测
TESTING
CNAS L2338

TEST REPORT

Report No：PWQC-WT-P24051621-1R

Sample Name ： Perovskite Photovoltaic cell

Client ： King Abdullah University of Science and Technology

Client Address ： Thuwal 23955-6900, Kingdom of Saudi Arabia

Type of Project ： Consignation

PHOTOVOLTAIC AND WIND POWER SYSTEMS QUALITY TEST CENTER, IEE, CHINESE ACADEMY OF SCIENCES

June, 19, 2024

PHOTOVOLTAIC AND WIND POWER SYSTEMS QUALITY TEST CENTER, IEE, CHINESE ACADEMY OF SCIENCES

Report No: PWQC-WT-P24051621-1R

Testing information:

Date：May, 16, 2024

Location：No.6 Bei-er-tiao, Zhongguancun, Haidian district, Beijing, China

Environmental conditions：(24.8±2)℃, (39.2±5)%RH

Testing items：

Measurement of photovoltaic current-voltage characteristics

Standards：

IEC 60904-1：2006 Photovoltaic (PV) devices
— Part 1：Measurement of photovoltaic current-voltage characteristics

Equipments：

Name	S/N	Expired date
Solar simulator	LE106-04	2024-10-15
Source Meter	LE177-01	2025-04-01
Reference cell	J-CH04	2025-04-06

Edited by(signatory)：Leekcu
Date：2024.6.19

Approved by(signatory)：Jiang Feifei
Date：2024.6.19

Page 1 of 5

PHOTOVOLTAIC AND WIND POWER SYSTEMS QUALITY TEST CENTER, IEE, CHINESE ACADEMY OF SCIENCES

Report No: PWQC-WT-P24051621-1R

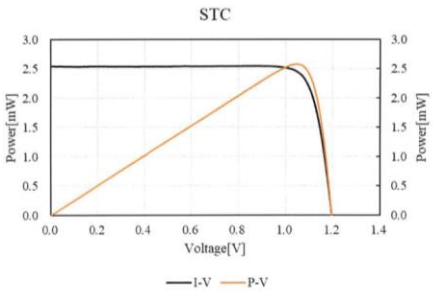

**Type**	Single junction perovskite solar cell
**Sample No.**	DC2024a062
**Area**	0.101 cm^2
**Isc**	2.543 mA
**Jsc**	25.181 mA/cm^2
**Voc**	1.196 V
**FF**	84.48 %
**Pm**	2.570 mW
**E_{ff}**	25.44 %
**Ipm**	2.471 mA
**Vpm**	1.040 V
**Voltage Sweep**	Reverse
**Sweep time**	7.6 s
**Temp**	25 ℃
**Irr**	100 mW/cm^2
**Curve**	A20240516151014

— End of Report —

Page 4 of 5

Fig. S19: Certified report results from an accredited photovoltaic certification institution (Photovoltaic and Wind Power Systems Quality Test Center, IEE, Chinese Academy of Sciences), the certificated efficiency is 25.44%.

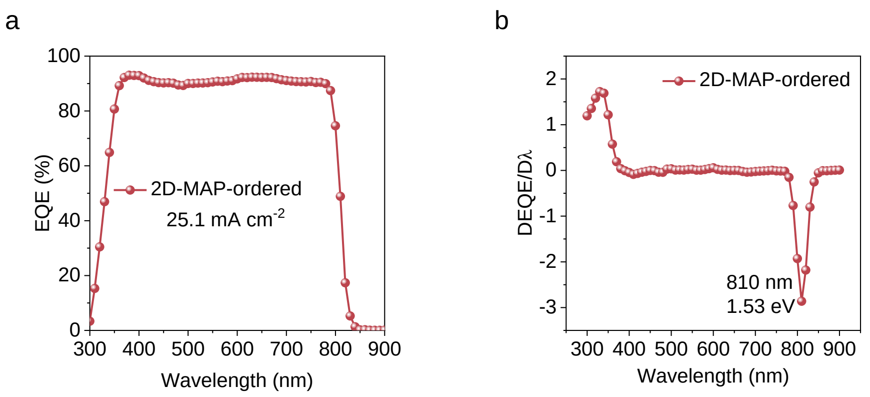

Fig. S20: **a**, EQE curve of 2D-MAP-ordered-based devices. **b**, First derivative of 2D-MAP-ordered-based device EQE.

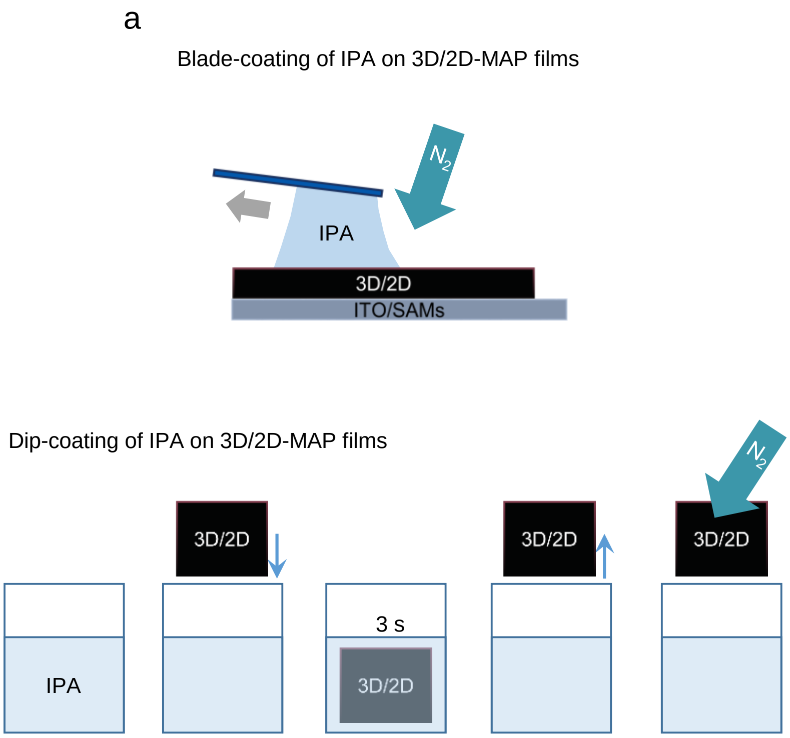

Fig. S21: Schematic diagram of blade-coating (**a**) and dip-coating (**b**) methods.

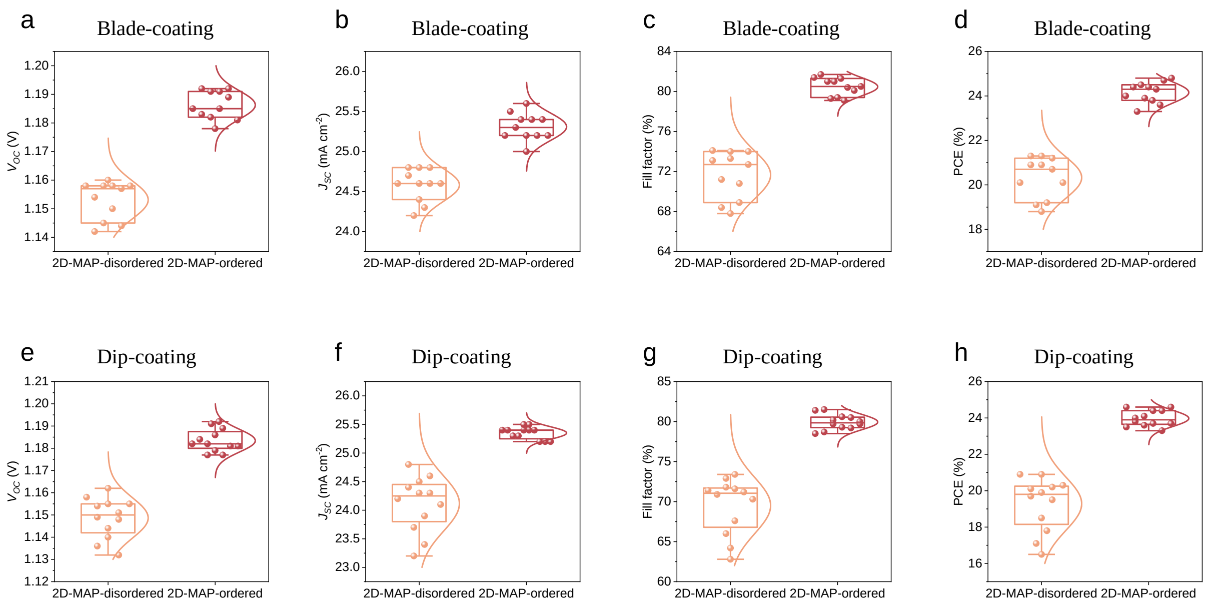

Fig. S22. Statistical comparison of photovoltaic parameters (V_{OC}, J_{SC}, FF, and PCE) of 2D-MAP-disordered and 2D-MAP-ordered-based devices by blade-coating (**a-d**) and dip-coating of IPA (**e-h**) on 3D/2D-MAP films (1 cm^2).

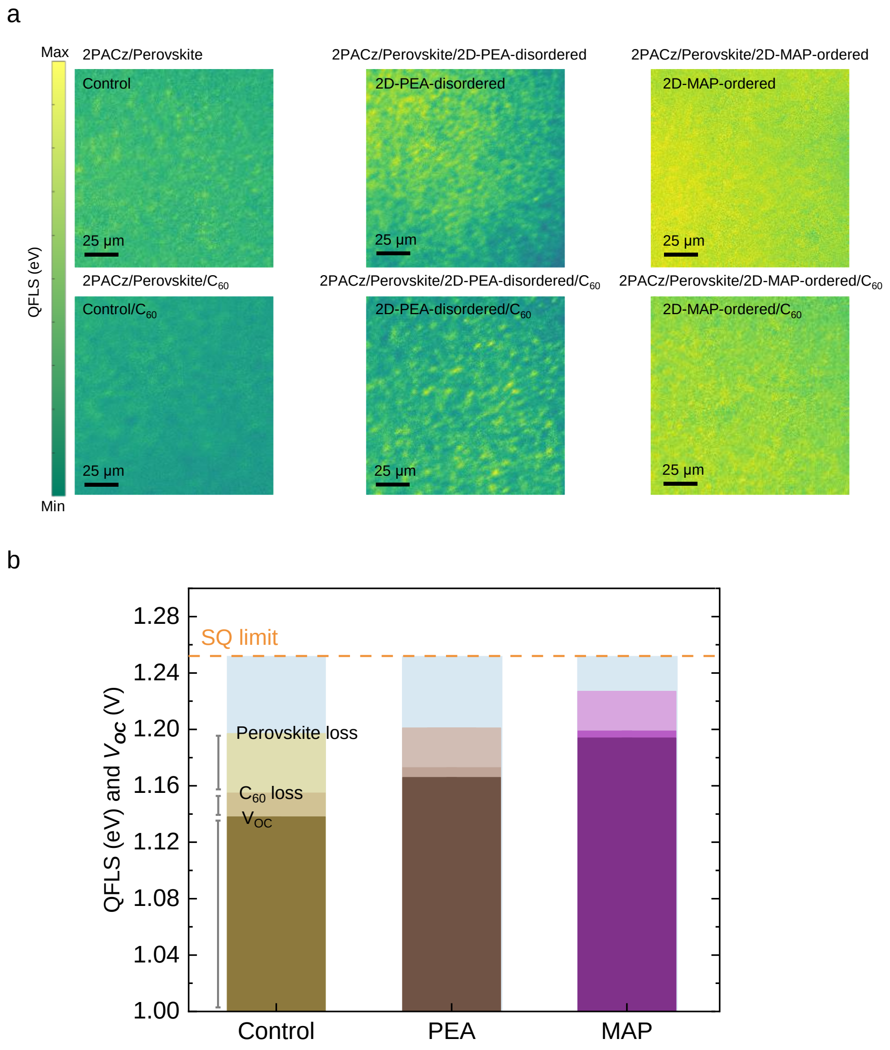

Fig. S23: a, QFLS mapping of perovskite films without and with C_{60}. **b**. QFLS and V_{OC} loss analysis.

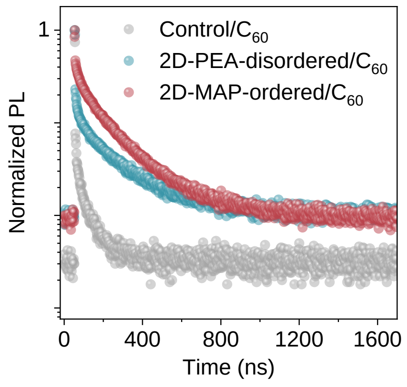

Fig. S24: Time-resolved PL (TRPL) spectra perovskite films.

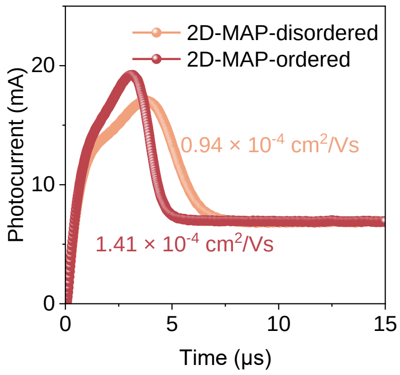

Fig. S25: Photo-CELIV transients of 2D-MAP-disordered and 2D-MAP-ordered-based devices.

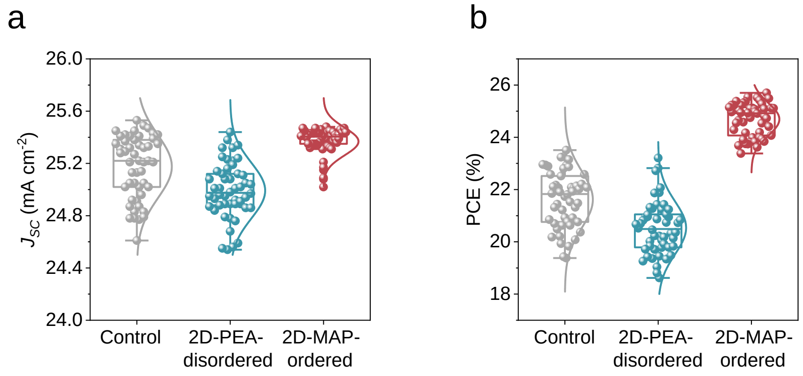

Fig. S26: Statistical comparison of photovoltaic parameters **a**, J_{SC} and **b**, PCE.

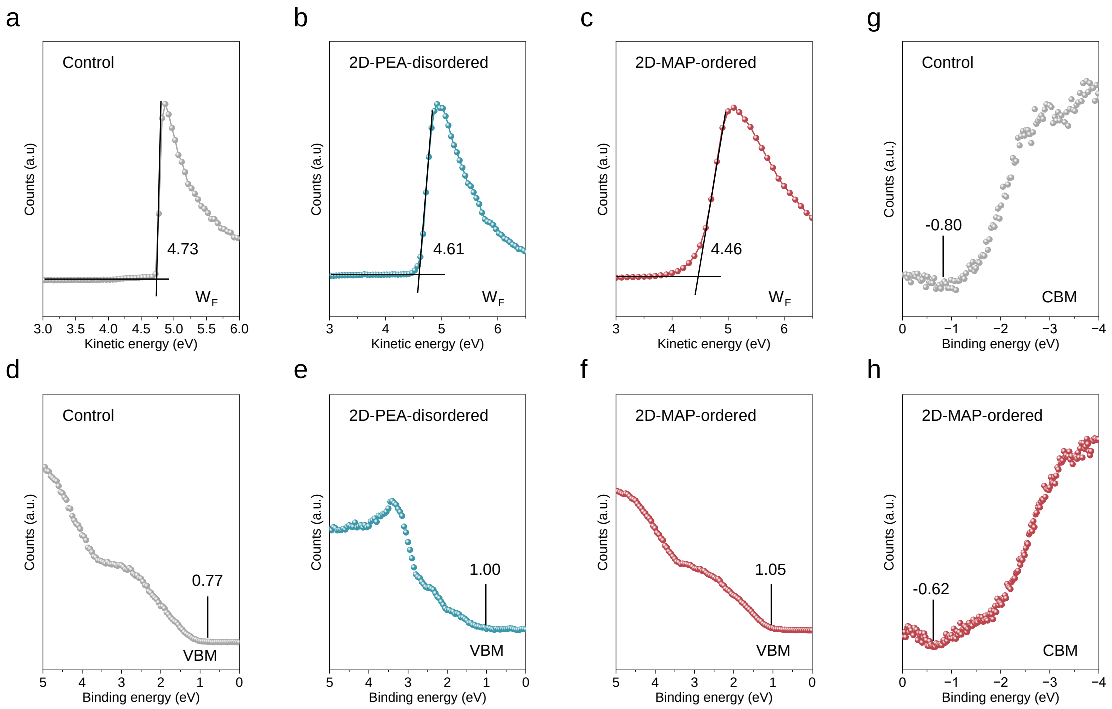

Fig. S27: a-f, UPS spectra of perovskite films. **g**,**h**, LE-IPES spectra of perovskite films. These spectrum portions show the secondary electron cutoff, valence region, and conduction region.

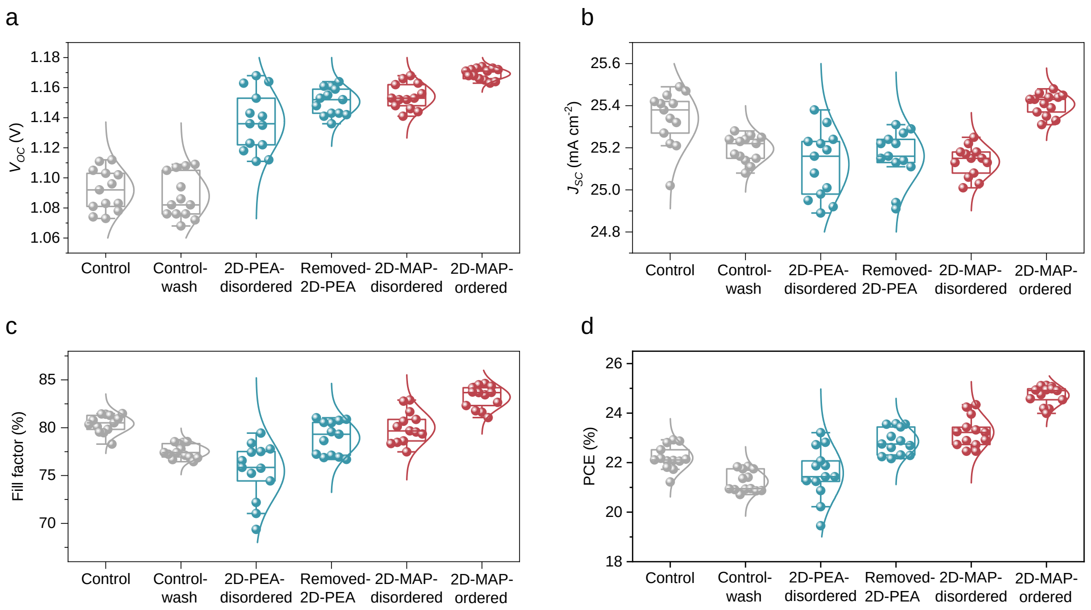

Fig. S28: a-d, Statistical comparison of photovoltaic parameters of with or without IPA post-dripping for control, PEA and MAP-based devices (V_{OC}, J_{SC}, FF, and PCE).

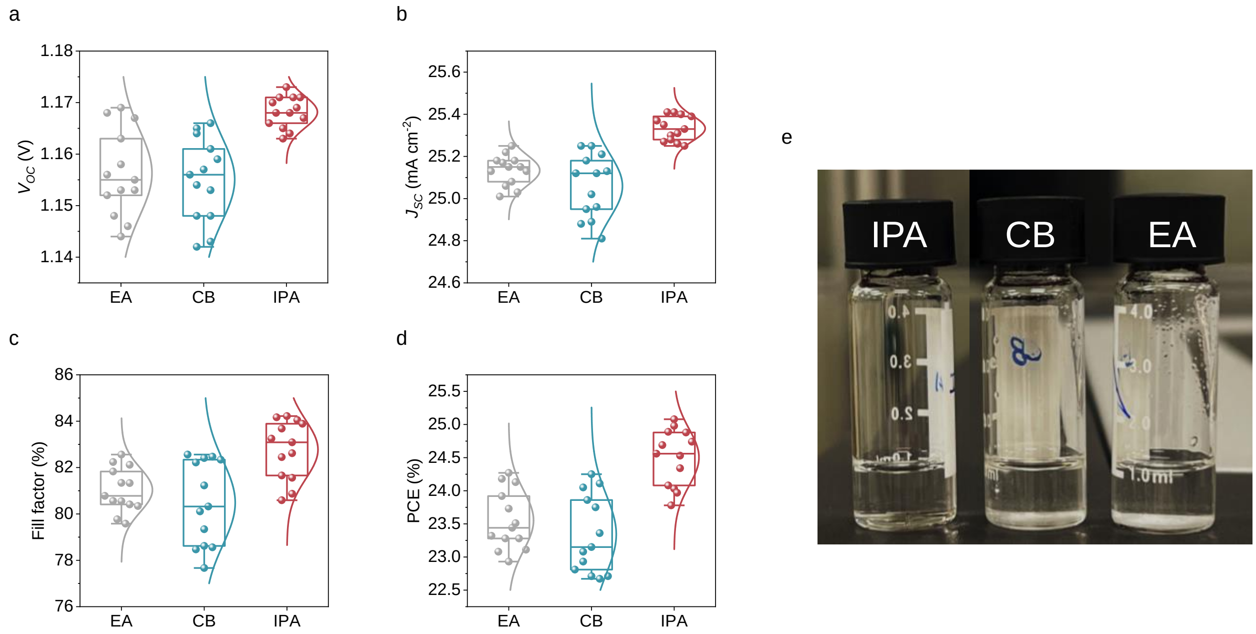

Fig. S29: a-d, Statistical comparison of photovoltaic parameters of with different solvent post-dripping for MAP-based devices (V_{OC}, J_{SC}, FF, and PCE). Ethyl acetate (EA); Chlorobenzene (CB); Isopropyl alcohol (IPA). **e**, Photograph images of MAPCl powders in different solvents.

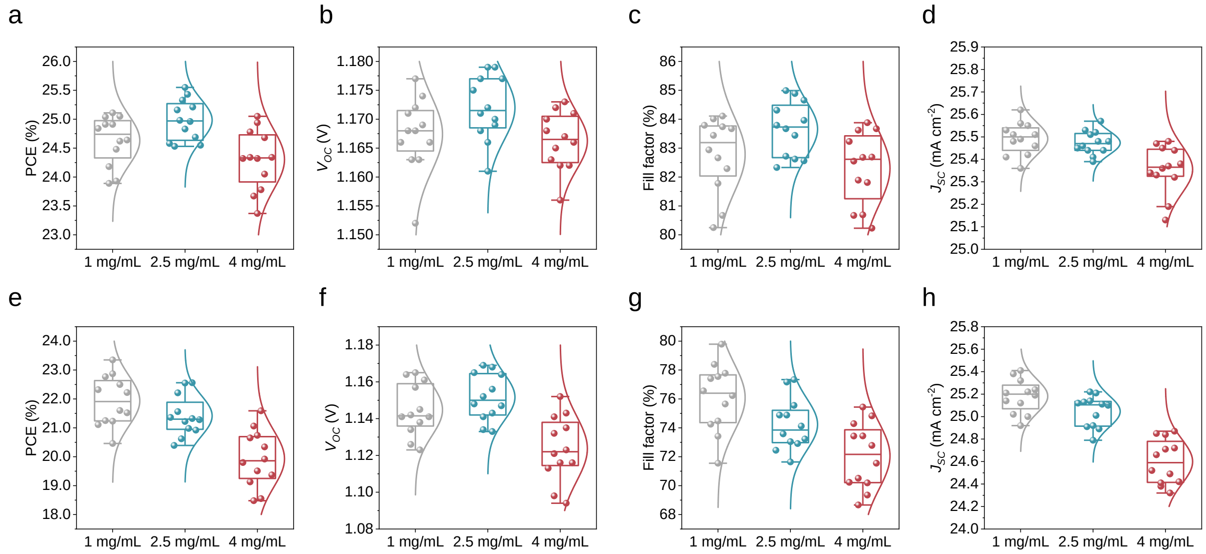

Fig. S30: PCE, V_{OC}, fill factor, and J_{SC} statistics of PSCs with different ligand concentrations of (**a-d**) 2D-MAP-ordered and (**e-h**) 2D-PEA-disordered. The statistics are obtained from 12 cells of each condition from different batches.

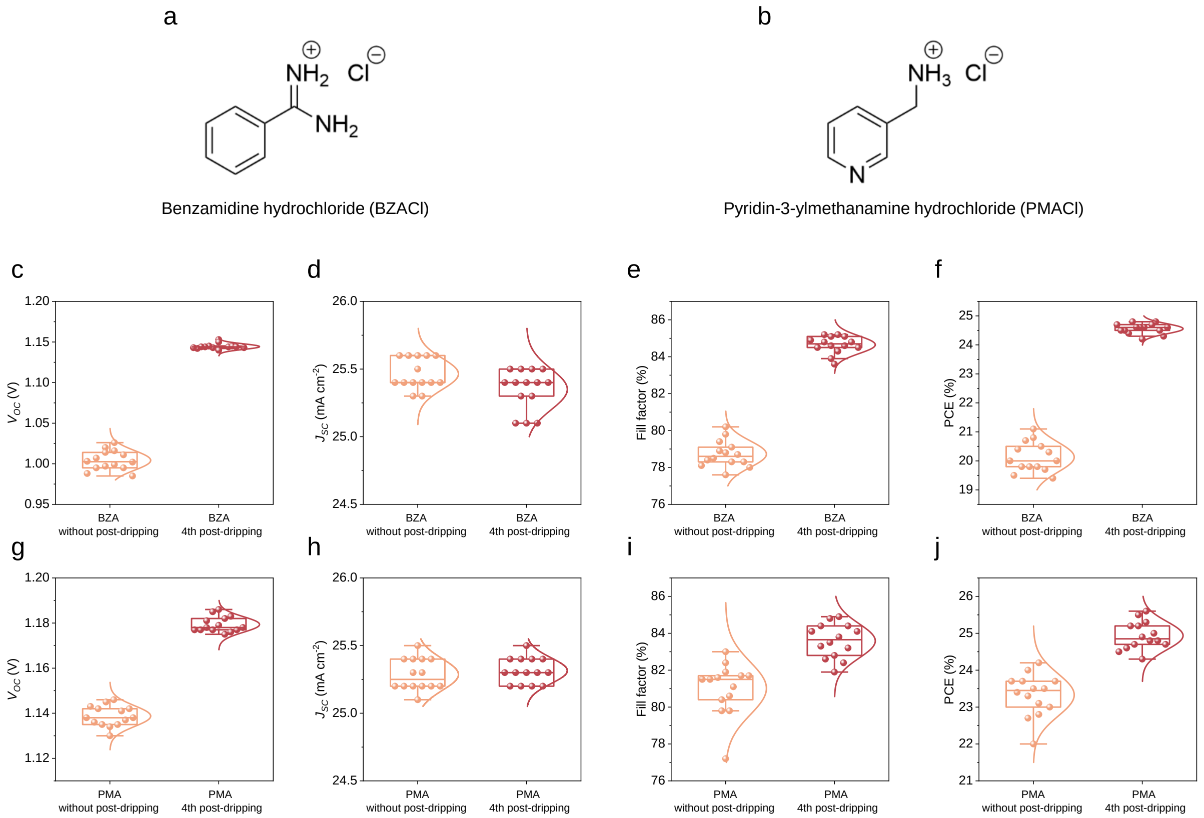

Fig. S31. a,b, Molecular structure of BZACl and PMACl. **c-j,** Statistical comparison of photovoltaic parameters (V_{OC}, J_{SC}, FF, and PCE) of BZA and PMA without post-dripping and BZA and PMA 4th post-dripping-based devices.

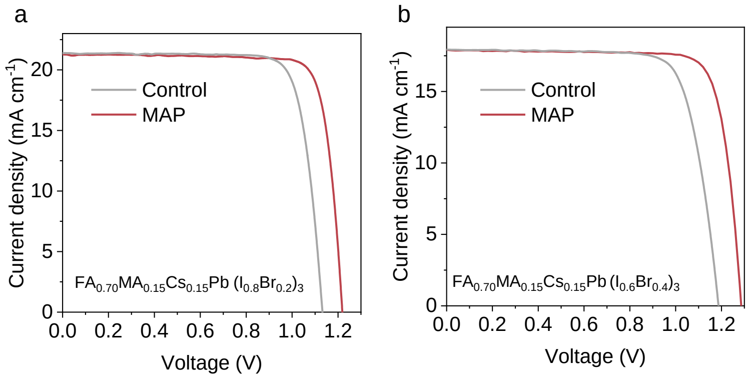

Fig. S32. *J-V* curves of the control and best-performing MAP-based devices with bandgaps of 1.68 eV (**a**) and 1.80 eV (**b**).

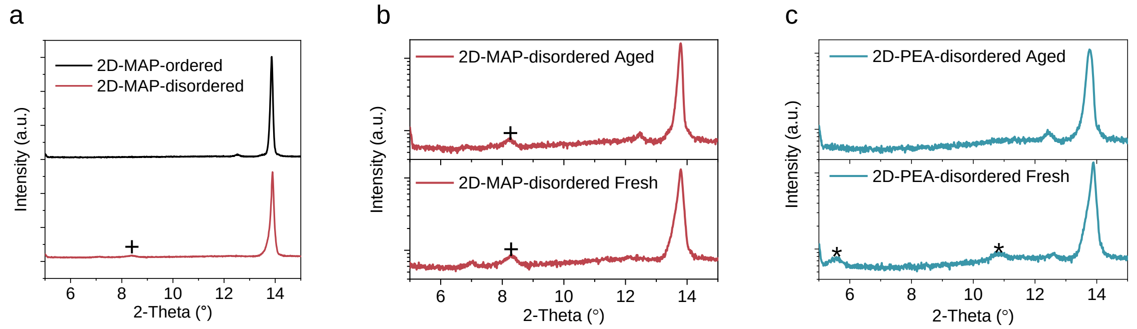

Fig. S33: a, XRD patterns of 2D-MAP-disordered and 2D-MAP-ordered-based perovskite films. **b**,**c**, XRD patterns of 2D-MAP-based and 2D-PEA-based perovskite films under thermal annealing at 85 °C for 0 hours and 48 hours. The black symbols (plus and asterisk) indicate the peak positions of MAP and PEA-based 2D perovskites, respectively. Conventional XRD equipment cannot detect the 2D perovskite peak on the surface after post-dripping due to its low resolution. Therefore, we employed XRD to analyze the degradation process using MAP and PEA samples without post-dripping.

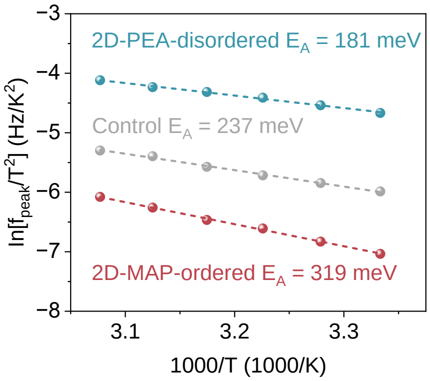

Fig. S34: Arrhenius plots of $\ln(f_{peak}/T^2)$ as a function of temperature were generated using the trap peak frequencies (f_{peak}) of the control, 2D-PEA-disordered and 2D-MAP-ordered-based devices, with the corresponding activation energies extracted.

Table S1. Champion devices performance of the control, 2D-PEA-disordered and 2D-MAP-ordered-based perovskite solar cells.

Samples	J_{SC} (mA cm^{-2})	V_{OC} (V)	FF (%)	PCE (%)
Control	25.35	1.138	81.45	23.51
2D-PEA-disordered	25.01	1.168	79.44	23.21
2D-MAP-ordered	25.53	1.195	85.5	26.05

Table S2. The lifetime statistics of perovskite/C_{60} films from TRPL.

Samples	τ_1 (ns)	A_1 (%)	τ_2 (ns)	A_2 (%)	τ_3 (ns)	A_3 (%)	τ_{ave} (ns)
Control/C_{60}	28.5	0.2858	108.3	0.2712	1.5	0.4429	38.18
PEA/C_{60}	36.7	0.1292	211.3	0.7795	2.1	0.0913	169.61
MAP/C_{60}	36.9	0.1533	184.3	0.8365	0.1	0.0101	159.82

Table S3. Device performance statistics of the control, 2D-PEA-disordered and 2D-MAP-ordered-based perovskite solar cells.

Samples	J_{SC} (mA cm^{-2})	V_{OC} (V)	FF (%)	PCE (%)
Control	25.19 ± 0.24	1.084 ± 0.039	79.54 ± 1.50	21.62 ± 1.08
2D-PEA-disordered	25.00 ± 0.23	1.128 ± 0.018	72.84 ± 2.95	20.53 ± 1.01
2D-MAP-ordered	25.37 ± 0.10	1.171 ± 0.008	83.11 ± 1.59	24.69 ± 0.63

Table S4. The energy level data of control, PEA and MAP-based perovskite films from UPS and IPES. *This CBM value is obtained using formula, $E_{VBM} = E_{CBM} - E_{bandgap}$ with the bandgap of 2D $(PEA)_2PbI_4$ perovskite is about 2.33 eV.[1]

Perovskites	WF (eV)	VBM (eV)	CBM (eV)
3D Control	4.73	5.50	3.93
2D-PEA (*n* = 1)	4.61	5.61	3.23*
2D-MAP (*n* = 1)	4.46	5.51	3.84

Table S5. Champion devices performance of control and MAP-based perovskite solar cells with bandgaps of 1.68 eV and 1.80 eV.

Devices		J_{SC} (mA cm^{-2})	V_{OC} (V)	FF (%)	PCE (%)
$FA_{0.70}MA_{0.15}Cs_{0.15}Pb(I_{0.8}Br_{0.2})_3$ (~1.68 eV)	Control	21.4	1.13	80.7	19.5
	MAP	21.3	1.22	82.8	21.5
$FA_{0.70}MA_{0.15}Cs_{0.15}Pb(I_{0.6}Br_{0.4})_3$ (~1.77 eV)	Control	17.9	1.19	77.1	16.4
	MAP	17.9	1.29	81.3	18.8

Supplementary References

1. Cho, K. T. *et al.* Selective growth of layered perovskites for stable and efficient photovoltaics. *Energy Environ. Sci.* **11**, 952-959 (2018).
