# Supplementary material for: Solvent-dripping modulated 3D/2D heterostructures for high-performance perovskite solar cells
Source: Nat Commun. 2025 Jan 26;16:1042. doi: 10.1038/s41467-025-56409-5 (PMC11763036; doi:10.1038/s41467-025-56409-5)
Supplement: Supplementary file 2 — Reporting Summary [file 41467_2025_56409_MOESM2_ESM.pdf]

Corresponding author(s): Thomas D. Anthopoulos

Solar Cells Reporting Summary

Nature Research wishes to improve the reproducibility of the work that we publish. This form is intended for publication with all accepted papers reporting the characterization of photovoltaic devices and provides structure for consistency and transparency in reporting. Some list items might not apply to an individual manuscript, but all fields must be completed for clarity.

For further information on Nature Research policies, including our data availability policy, see Authors & Referees.

Experimental design

Please check: are the following details reported in the manuscript?

1. Dimensions

Item	Yes	No	Response
Area of the tested solar cells	☒	☐	The area of tested cells is 0.063 cm square
Method used to determine the device area	☒	☐	Shadow mask

2. Current-voltage characterization

Item	Yes	No	Response
Current density-voltage (J-V) plots in both forward and backward direction	☒	☐	We provide both forward and backward direction scans in Figure 3b
Voltage scan conditions<br>*For instance: scan direction, speed, dwell times*	☒	☐	Experimental methods
Test environment<br>*For instance: characterization temperature, in air or in glove box*	☒	☐	Experimental methods
Protocol for preconditioning of the device before its characterization	☐	☒	No preconditoning protocol was used.
Stability of the J-V characteristic<br>*Verified with time evolution of the maximum power point or with the photocurrent at maximum power point; see ref. 7 for details.*	☒	☐	We provide 200 s maximum power point tracking in Figure 3c to evaluate the stabilization PCE.

3. Hysteresis or any other unusual behaviour

Item	Yes	No	Response
Description of the unusual behaviour observed during the characterization	☐	☒	No unusual behavior and negligible hysteresis were observed.
Related experimental data	☐	☒	We provide J-V hysteresis scan in Figure 3b

4. Efficiency

Item	Yes	No	Response
External quantum efficiency (EQE) or incident photons to current efficiency (IPCE)	☒	☐	We provide EQE data in Figure S18
A comparison between the integrated response under the standard reference spectrum and the response measure under the simulator	☒	☐	Figure 3b and S18
For tandem solar cells, the bias illumination and bias voltage used for each subcell	☐	☒	*Explain why this information is not reported/not relevant.*

5. Calibration

Item	Yes	No	Response
Light source and reference cell or sensor used for the characterization	☒	☐	Experimental methods
Confirmation that the reference cell was calibrated and certified	☒	☐	Our solar simulator was calibrated by KG-5 solar cells (Certified by Newport)

Calculation of spectral mismatch between the reference cell and the devices under test

☒ Yes ☐ No

A spectral mismatch was performed based on the spectral intensity of the solar simulator and EQEs of our devices. The mismatch current density value is about 1.1%. Main Text and Figure S18.

6. Mask/aperture

Size of the mask/aperture used during testing

☒ Yes ☐ No

a black metal mask with an aperture area of 0.063 cm square was used to define the active area of the devices.

Variation of the measured short-circuit current density with the mask/aperture area

☒ Yes ☐ No

Not significant variations were observed.

7. Performance certification

Identity of the independent certification laboratory that confirmed the photovoltaic performance

☒ Yes ☐ No

Figure S17

A copy of any certificate(s)
Provide in Supplementary Information

☒ Yes ☐ No

Figure S17

8. Statistics

Number of solar cells tested

☒ Yes ☐ No

Figures 3i,j, Figure. S24 and table S3

Statistical analysis of the device performance

☒ Yes ☐ No

Figures 3i,j, Figure. S24 and table S3

9. Long-term stability analysis

Type of analysis, bias conditions and environmental conditions
For instance: illumination type, temperature, atmosphere humidity, encapsulation method, preconditioning temperature

☒ Yes ☐ No

Figure 4
